# Efficacy, Safety, and Regulation of Cannabidiol on Chronic Pain: A Systematic Review

**DOI:** 10.7759/cureus.26913

**Published:** 2022-07-16

**Authors:** Maria Resah B Villanueva, Narges Joshaghani, Nicole Villa, Omar Badla, Raman Goit, Samia E Saddik, Sarah N Dawood, Ahmad M Rabih, Ahmad Niaj, Aishwarya Raman, Manish Uprety, Maria Calero, Safeera Khan

**Affiliations:** 1 Internal Medicine/Family Medicine, California Institute of Behavioral Neurosciences and Psychology, Fairfield, USA; 2 Psychiatry and Behavioral Sciences, California Institute of Behavioral Neurosciences and Psychology, Fairfield, USA; 3 Internal Medicine, California Institute of Behavioral Neurosciences and Psychology, Fairfield, USA; 4 General Surgery, California Institute of Behavioral Neurosciences and Psychology, Fairfield, USA; 5 Pediatrics, California Institute of Behavioral Neurosciences and Psychology, Fairfield, USA; 6 Obstetrics and Gynecology, California Institute of Behavioral Neurosciences and Psychology, Fairfield, USA

**Keywords:** chronic pain, cannabis, hemp, cbd, cannabidiol

## Abstract

We conducted a systematic review to determine the efficacy and safety of cannabidiol (CBD) for chronic pain. The systematic review is according to the Preferred Reporting Items for Systematic Review and Meta-Analysis (PRISMA) 2020 checklist.

Five databases (PubMed, PubMed Central, Medline, Cochrane Library, and ScienceDirect) were searched using cannabidiol, CBD, hemp, and chronic pain. Inclusion criteria used were studies on adult populations >18 years old; pain symptoms >three months duration; all available preparations of CBD; human studies only; publication in English in the past five years. A total of 2298 articles were found. Inclusion criteria were applied, and quality assessments were done, resulting in 12 publications eligible for the review.

CBD and tetrahydrocannabinol (THC), both from Cannabis plants with almost identical chemical structures, attach to the CB receptor, eliciting different effects like the psychoactivity seen on THC but less or none in CBD. Regulations of CBD worldwide differ from each other due to the insufficiency of solid evidence to establish its benefit versus the risks. However, a few studies are showing the benefits of CBD not only for chronic pain but also for sleep improvement and quality of life.

In conclusion, CBD is an excellent alternative to an opioid in chronic pain because CBD is non-intoxicating in its pure form. More clinical trials should be done to prove CBD's significance clinically and statistically.

## Introduction and background

According to Forbes, in October 2020, cannabidiol (CBD) sales in the United States reached $4.2 billion after the federal government legalized hemp-derived CBD in 2018 [1]. In addition, the World Health Organization (WHO) in 2019 re-classified CBD and <0.2% of delta-9-tetrahydrocannabinol (THC) as not under international control and recognized its medical value in 2020 [2]. Hence, CBD is a rapidly expanding business expected to increase its value to $20 billion in 2025 [1].

CBD is a nonintoxicating chemical ingredient from the Cannabis sativa plant [3]. CBD's medical value was a hot topic for debate before being recognized in the medical field. One preparation of CBD approved by the U.S. Food and Drug Administration (FDA) is Epidiolex, an oral solution given to patients less than two years old to treat two rare and severe forms of seizure, Lennox-Gastaut syndrome and Dravet syndrome [4]. In addition, dronabinol [a synthetic delta-9-tetrahydrocannabinol (THC) product] and nabilone (like THC) were regulated by the FDA for the treatment of chemotherapy-induced nausea and vomiting [5]. Dronabinol is also used for AIDS-associated anorexia. With its federal legalization, CBD dispensaries continue to open one after another. People have more access to a wide variety of CBD products like cannabis flowers, tinctures, concentrates, topical lotion/creams, and edibles which are self-administered and with little or no supervision by a physician [6]. CBD oils provide relief for various conditions, including pain without intoxication [3]. Regulations of cannabis products remain a challenge for most countries.

Chronic pain is a continuous or recurring pain for three months or longer experienced by a patient due to various causes. Different types of chronic pain are identified based on their nature, location, and characteristics. It is a significant cause of disability globally, and billions of dollars are spent annually to alleviate its outcomes [7]. While the opioid crisis increases, CBD's role in pain management unveils as animal studies show promising evidence [8]. Further investigation and trials into CBD's therapeutic value are ongoing due to its natural source, numerous usages, lower risk of addiction or dependency, and relative safety [7]. FDA regulation of CBD needs more clinical trials to determine its effectiveness and safety and should meet proper standards for authorization [9].

This paper aims to answer the efficacy and safety of CBD in chronic pain using a systematic review of articles from five databases. This study will fill the existing gap and update knowledge on CBD's role in chronic pain.

## Review

Methods

Protocol

This descriptive systematic review was done according to the Preferred Reporting Items for Systematic Review and Meta-Analysis (PRISMA) 2020 checklist [10]. Before the search of the databases, a protocol was made and shared with the research team to analyze and finalize. The main question of the review: What is the efficacy and safety of CBD in adult patients with chronic pain? The PICO strategy was used to formulate the question of this review. The review protocol can be acquired with a request addressed to the lead author.

Search Strategy

PubMed, PubMed Central (PMC), Medline, Cochrane Library, and ScienceDirect were utilized as the major databases and search engines. In PubMed, the search was done using keywords and a medical subject heading (MeSH). The keywords "Cannabidiol" and "chronic pain" were applied to obtain related literature. The MeSH strategy used in PubMed and PMC were: ("Cannabidiol/adverse effects"[Majr] OR "Cannabidiol/isolation and purification"[Majr] OR "Cannabidiol/metabolism"[Majr] OR "Cannabidiol/pharmacokinetics"[Majr] OR "Cannabidiol/pharmacology"[Majr] OR "Cannabidiol/poisoning"[Majr] OR "Cannabidiol/therapeutic use"[Majr] OR "Cannabidiol/toxicity"[Majr]) AND ("Chronic Pain/drug therapy"[Mesh] OR "Chronic Pain/prevention and control"[Mesh] OR "Chronic Pain/therapy"[Mesh]). Booleans "AND" and "OR" were used.

Additionally, keywords such as Cannabidiol, CBD, Hemp, Marijuana, Chronic Pain, and other synonyms were applied to the other databases. Furthermore, other publications in the reference list and related studies were also examined to see if they were relevant and could be included in this review.

There were a total of 2298 articles extracted from all the databases. PubMed, PMC, and Medline have 289 articles. The Cochrane Library and Science Direct gave 73 and 1936 articles, respectively. The databases were last accessed on April 2022.

Eligibility Criteria

A PRISMA flow diagram 2020 was used to show the study’s inclusion and exclusion of articles found in the databases used. The inclusion criteria for eligibility were: (i) studies in an adult population >18 years old; (ii) patients with pain symptoms of less than three months duration; (iii) all available preparations of CBD; (iv) human studies only; (v) publication in English; and (vi) publication in the last five years. Studies with pediatric patients, acute pain, and animal studies were excluded. Studies with no available full text were also excluded from the review.

Data Collection Process: Synthesis, Extraction, and Management

All titles of the articles initially obtained from databases were selected by applying the eligibility criteria set. Duplicates were eliminated. The titles were read, and unrelated articles were excluded. The abstracts of the remaining articles were further screened for relevance. The full text of the publications left was obtained, and those without full text were excluded.

Quality Assessment in Included Studies

The Scale for the Assessment of Narrative Review Articles (SANRA) [11], Assessment of Multiple Systematic Reviews (AMSTAR) [12], JBI tool for Case Reposts [13], New Castle Ottawa [14], and Risk of Bias 2 [15] in the Cochrane Risk Assessment Tool (RoB 2) were used to identify the eligible articles based on the kind of study for each publication. Two co-authors (NJ and NV) assessed the eligibility of the articles.

Results

Search Results

**Figure 1:**
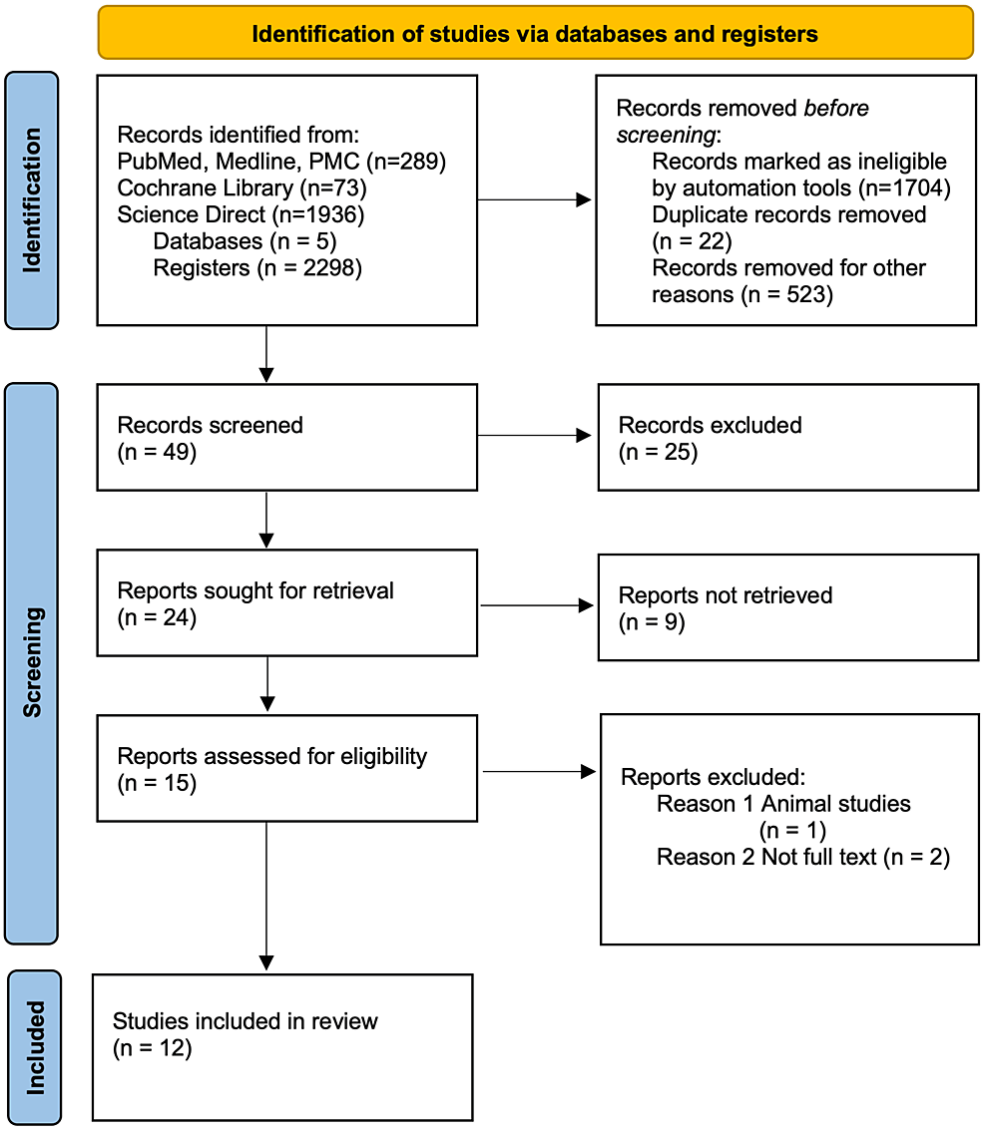
PRISMA 2020 flow diagram for new systematic reviews From: Page et al. The PRISMA 2020 statement: an updated guideline for reporting systematic reviews. BMJ 2021;372:n71. doi: 10.1136/bmj.n71 [10]

Five databases (PubMed, PubMed Central, Medline, Cochrane Library, and ScienceDirect) were used to identify publications included in the review. Figure 1 is a PRISMA 2020 flow diagram showing how related studies included in the review were identified [10].

Using MeSH and keywords like cannabidiol, CBD, hemp, and chronic pain, 2298 publications were obtained. PubMed, PMC, and Medline have 289 publications. The Cochrane Library and Science Direct listed 73 and 1936 publications, respectively. A preliminary screening was done. Upon checking for duplicates, 22 publications were excluded. Filters were applied using the exclusion and inclusion criteria to exclude 1704 publications. Other reasons include manual screening and protocol articles that eliminated 523 publications. Abstracts were screened, and 25 publications out of 49 were excluded. Fifteen full papers were retrieved, while nine were not. Two do not have full texts, and one is an animal study, hence excluded. The remaining publications were assessed for eligibility using the appropriate assessment tool. A total of 12 studies were found eligible for this review.

Results of Quality Appraisal

A summary of the studies and the quality appraisal tool used for each one is shown in Table 1.

**Table 1:** Overview of the publications and the corresponding quality assessment tool SANRA: Scale for the Assessment of Narrative Review Articles, AMSTAR: Assessment of Multiple Systematic Reviews, JBI: Joanna Briggs Institute, RoB: Risk of Bias

Kind of study	Quality assessment tool	Number of articles
Review	SANRA	5
Systematic review	AMSTAR	3
Case report	JBI tool	1
Observational	New Castle Ottawa	2
Randomized controlled trial	Cochrane Bias Assessment tool (RoB 2)	1

The study must get a 70% to be eligible for this review. Detailed quality appraisals with the corresponding tools used for each study are shown below. Table 2 shows the use of SANRA for five review articles.

**Table 2:** SANRA quality assessment tool SANRA: Scale for Assessment of Narrative Review Articles [11]

Publication	Boyagi et al. [8]	Mauer et al. [5]	VanDolah et al. [3]	Mücke et al. [16]	Fisher et al. [17]
Justification of the article's importance in the readership	2	2	2	2	2
Statement of concrete aims or formulation of questions	1	1	1	2	1
Description of the literature search	2	2	2	2	2
Referencing	2	2	2	2	2
Scientific reasoning	2	2	2	2	2
Appropriate presentation of data	1	2	2	2	2

AMSTAR is utilized to assess the eligibility of three systematic reviews shown in Table 3.

**Table 3:** AMSTAR 2 quality assessment tool AMSTAR: Assessment of Multiple Systemic Reviews [12]

Publication	Rabgay et al.[18]	Pagano et al. [19]	Scuteri et al. [20]
Did the research questions and inclusion criteria for the review include the components of PICO?	Y	Y	Y
Did the report of the review contain an explicit statement that the review methods were established prior to the conduct of the review, and did the report justify any significant deviations from the protocol?	Unclear	Unclear	Unclear
Did the review authors explain their selection of the study designs for inclusion in the review?	Y	Y	Y
Did the review authors use a comprehensive literature search strategy?	Y	Y	Y
Did the review authors perform study selection in duplicate?	Y	Y	Y
Did the review authors provide a list of excluded studies and justify the exclusions?	Unclear	Unclear	Unclear
Did the review authors describe the included studies in adequate detail?	Y	Y	Y
Did the review authors use a satisfactory technique for assessing the risk of bias (RoB) in individual studies that were included in the review?	Y	Y	Y
Did the review authors report on the sources of funding for the studies included in the review?	Y	Y	Y
Did the review authors account for RoB in individual studies when interpreting/discussing the results of the review?	Unclear	Y	y
Did the review authors provide a satisfactory explanation for, and discussion of, any heterogeneity observed in the results of the review?	Y	Y	Y

Table 4 illustrates JBI as a quality assessment tool for case reports.

**Table 4:** JBI quality assessment tool for case report JBI: Joanna Briggs Institute [13]

Publication	Diaz et.al. [21]
Demographic characteristics	Y
History and timeline	Y
Presentation of clinical condition	Y
Diagnostic test and results	Y
Intervention and treatment	Y
Post-intervention clinical condition	Y
Adverse events	N
Take-away lessons	Y

New Castle Ottawa Tool is used to evaluate the eligibility of two observational studies in Table 5.

**Table 5:** New Castle Ottawa quality assessment tool for observational studies [14] *Indicates a yes as the answer

Publication	Capano et.al. [7]	Boehnke et.al. [6]
Representativeness of the exposed cohort	*	*
Selection of the non-exposed cohort		
Ascertainment of exposure	*	*
Demonstration that outcome of interest was not present at start of study	*	*
Comparability of cohorts on the basis of the design or analysis	*	*
Assessment of outcome	*	*
Adequacy of follow up of cohorts	*	*

The RoB 2 tool is a revised Cochrane RoB employed for RCT assessment as shown in Table 6.

**Table 6:** RoB 2 quality assessment tool for RCT RoB: risk of bias [15]; RCT: randomized clinical trial

Publication	Lichtman et al. [22]
Randomization process	Low
Deviations from the intended interventions (effect of assignment to intervention)	Low
Missing outcome data	Low
Measurement of the outcome	Low
Selection of the reported result	Low
Overall risk of bias	Low

Data Extraction

A total of 12 publications were found eligible for this systematic review. Each article included in this review was read and scrutinized. Relevant information was summarized in Table 7 to show an overview of each study collected from the databases.

**Table 7:** Summary table for the included Studies CBD: cannabidiol; ROS: reactive oxygen species; THC: tetrahydrocannabinol; RCT: randomized clinical trial

Author and year of publication	Purpose of the study	Number of patients/studies	Type of study	Main findings
Boyaji et al. [8]	To find an alternative treatment that is safer and more effective than opioids to combat chronic pain challenges.	7 studies	Review	Cannabidiol is a promising alternative to manage pain but hard to make recommendations due to the difficulty of attributing the therapeutic properties to CBD alone.
Fischer et al. [17]	To identify new scientific advances to make an updated 'Lower Risk Cannabis Use Guideline' (LRCUG).	Not specified	Review	The high-risk group (early adolescent, patient with comorbidity, and pregnant or breastfeeding women) can have a harmful outcome from CBD use; hence, lowering the risk factor can also lessen the adverse outcome.
Mauer et al. [5]	To know the safety, efficacy, and adverse effect of cannabis-based products on athletes.	2224 patients	Review	Recommendations from physicians are promising but hard to do since studies available are from non-athletic subjects.
VanDolah et al. [3]	To identify a non-intoxicating alternative to opioids in chronic pain management.	102 studies	Review	CBD and hemp oil have a positive potential benefit in managing chronic pain, and more research is required.
Mücke et al. [16]	To compare if cannabis-based medication versus placebo or conventional drugs are safe, efficient, and tolerable.	16 studies, 1750 patients	Review	Some patients with neuropathic pain may benefit from cannabis-based medicine (3rd or 4th line therapy), and no high-quality evidence to show how efficacious cannabis-based drugs are.
Pagano et al. [19]	To evaluate the safety level, dosing, and timing of CBD on healthy cells.	29 studies	Systemic review	Dose-dependent inhibition of cell viability above two micrograms while apoptosis is observed in 10 micrograms CBD. Anti-inflammatory effects and decreased ROS production were also noted.
Rabgay et al. [18]	To determine the role of the route of administration of cannabis and cannabinoids on pain and its side effects.	25 studies, 2270 patient	Systemic review	Among different routes of administration of THC/CBD, the Oro-mucosal route was dominant in controlling pain from different causes like cancer, neuropathic, and nociceptive pain.
Scuteri et al. [20]	To know the efficacy of cannabinoid-based products in ocular pain regimens.	4 studies	Systemic review	Preclinical studies are needed to establish the efficacy of CBD in ocular inflammation and neuropathic pain, although analgesia is observed using CBD oil. It is noted that the is analgesia as well on the topical formulation.
Diaz et al. [21]	To describe a patient with chronic pressure injury treated with medical cannabis oil (THC and CBD) for pain relief and sleep improvement.	1 patient	Case report	Medical Cannabis oil containing THC and CBD taken orally improves pain and sleep with direct or indirect effect on wound healing.
Boehnke et al. [6]	To describe naturalistic cannabis use routine and its benefits.	1087 patients	Observational (cross-sectional)	The risk and benefits of medical cannabis can be further observed when administration route profiles are used to make subgroups.
Capano et al. [7]	To determine the effect of CBD (full hemp extract) on chronic pain regarding the quality of life and opioid use.	131 patients	Observational (prospective cohort)	CBD improves pain, quality of life and sleep quality and decreases opioid use in patients who have chronic pain on narcotics.
Lichtman et al. [22]	To assess the use of nabiximols as an adjunct to opioids in advanced cancer patients with poorly controlled pain.	397 patients	RCT	Advanced cancer patients on lower opioid therapy with early intolerance to opioid may benefit more from CBD as adjunct medication, although CBD is not superior to placebo on primary efficacy.

Discussion

CBD is a fast-growing business following its federal legalization in 2018. With this, more people have gained access to CBD, especially those with chronic pain on pain medications, and have experienced promising outcomes. Hence, more research and studies are being done to give patients with chronic pain an efficacious and safe alternative to the existing kinds of pain medication available on the market.

Cannabidiol versus Tetrahydrocannabinol

**Figure 2:**
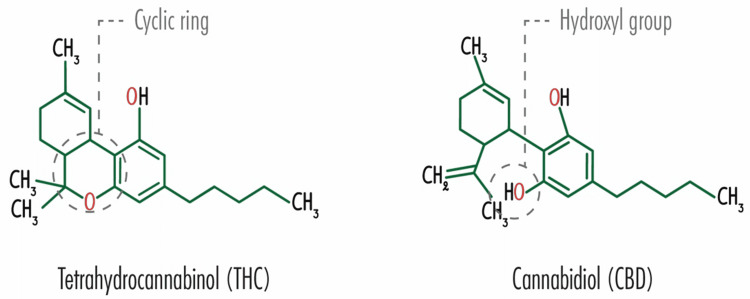
Chemical structure of tetrahydrocannabinol and cannabidiol Source: Analytical Cannabis: CBD vs THC – What are the Main Difference? with permission [23]

The Cannabis sativa plant has many strains, but the more popular ones are marijuana and hemp. Phytocannabinoids can be extracted from the cannabis plant, and this active chemical, when combined with the receptor, affects the functioning of the body in many ways. THC and CBD are famous examples of these phytocannabinoids obtained from marijuana and hemp, respectively. THC attaches to cannabinoid receptor 1 (CB1) while CBD attaches to several receptors like CB receptors, transient receptor potential vanilloid 1, G protein-coupled receptor 55, and serotonin 5-HT1A [3]. CBD and THC have the same molecular formula, C21H30O2, and an almost identical molecular mass of 314.464 g/mol and 314.469 g/mol, respectively [23]. Figure 2 illustrates the structural formulas of CBD and THC, highlighting a vital difference between the two: a cyclic ring for THC and a hydroxyl group for CBD.

This difference makes THC a potential partial agonist to the CB1 receptor and CBD a negative allosteric modulator, on the other hand [23]. The stimulation of CB1 receptors produces the psychotropic effects experienced with THC consumption but is not evident in CBD use. Metabolism is by the cytochrome P450 superfamily; hence many drug interactions are possible.

In a review done by VanDolah et al., more studies focused on the benefits of prescribed THC drugs; on the other hand, four studies were linked to CBD’s potential therapeutic actions, safety, and adverse effects [3]. Some of the potential therapeutic actions of CBD include relief of chronic pain, sleep disorders, spasticity and Tourette syndrome, nausea and vomiting in chemotherapy, and weight gain in HIV patients, to name a few. Its adverse effects include liver toxicity, somnolence, decreased appetite, diarrhea, and low blood pressure [3]. In addition, Scuteri et al., a systematic review of four studies, revealed that CB2 agonist HU308 alleviates inflammation in the eyes by reducing uveitis-induced leukocyte adhesion and lipidome profile changes [20]. It also highlights the antinociceptive and anti-inflammatory effects of D8-THC, cannabidiol, derivative HU308, and the new racemic CB1 allosteric ligand [20]. Another study with 2224 patients by Maurer et al. revealed that the patients’ post-injury three and four-week use of cannabis after concussions resulted in a lower severity score but not faster recovery from concussion symptoms [5]. The case report of Diaz et al. on a patient with pressure injury exhibiting pain and sleep problems was given with three different medical cannabis oils (1 CBD-dominant and 2 THC-dominant) in increasing doses and revealed an improvement in sleep quality with a decrease in pain and anxiety [21]. An incidental wound improvement was noticed starting at two weeks post-treatment [21]. These studies highlighted different benefits of CBD on different areas of the body, making the potential value of the CBD product even greater. The studies complement each other in strengthening the value of CBD medically when used on different body parts.

Regulation on Cannabis

**Figure 3:**
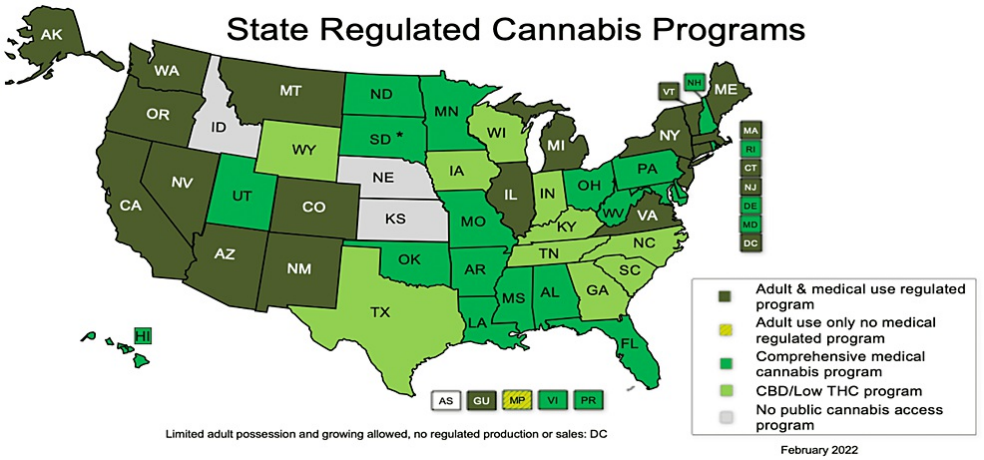
Cannabis regulation per U.S. states and territories Source: National Conference of State Legislatures with permission [24]

In the 2014 Agricultural Act, hemp and marijuana differences are notable, defining the legality of "industrial hemp" (*Cannabis sativa L*.) and any parts of the plant (with THC content <0.3% dry weight) for research purposes [3]. The use of medical cannabis is permitted in 37 states, four territories, and the District of Columbia and is prohibited in three states and one territory [24]. Figure 3 shows a clear picture of the regulation of cannabis per state in the United States.

With more states opening their doors to the medical benefit of CBD, the issue of obtaining good quality CBD poses a risk for those who want to use it as an alternative to their current pain medications [25]. There is a high price tag on good quality CBD available, and affordable CBD products are not 100% reliable due to some manufacturers' mislabeling issues about their exact content. In addition, the FDA still cannot impose strict regulations because CBD is not considered a pharmaceutical agent anymore [9].

Efficacy and Safety of CBD

In comparison to THC, CBD is a relatively new drug, and studies are limited to establishing its safety and efficacy. Moreover, the regulations surrounding the use of CBD are still highly debatable. In a systematic review of 229 studies done by Pagano et al., the effects of CBD on healthy cell characteristics such as cell viability, cell proliferation, wound repopulation, apoptosis, and cell cycle were tackled [19]. Dose-dependent administration showed a significant reduction of cell viability (above 2 mM); oral cells are inhibited at 10 mM, while cell proliferation inhibition is evident in all doses used (2, 6, and 10 mM). Cell migration decreased after giving 10 mM for 24 hours [19]. However, there was no significant change at 6 mM. Lastly, an increase in apoptosis is observed at 10 mM [19]. These observations show that a variable amount of CBD exerts different effects on a healthy cell. The dosage mainly dictates the extent of the results. It can be noted that a higher dose means more inhibition of cell processes but more stimulation of apoptosis.

Furthermore, Rabgay et al. conducted a systematic review of 25 studies with 2270 patients regarding the different dosages and routes of administration for CBD [18]. They found out that cannabis and cannabinoids act on different types of pain depending on the dosage and route of administration. A low dose for pain relief was used for all studies reviewed and exhibited an average dose of 19.82 mg/day [18]. Furthermore, they discovered that the difference in the dosage administered elicited relief in different pain types, such as neuropathic pain, which is 23.56 mg/day, cancer pain, which is 19.69 mg/day, and nociceptive pain, which is 13.75 mg/day [18]. In addition, different routes of administration showed other forms of pain relief. The oromucosal route is THC/CBD and THC for neuropathic and cancer pain; the oral route is THC for cancer pain; and the inhalation of standardized cannabis with THC (SCT) for neuropathic and oral standardized cannabis extract with THC (SCET) for nociceptive pain [18]. Rabgay et al. concluded that there is no sufficient evidence to fully establish CBD’s efficacy on pain. In a review done by Boyaji et al. on seven studies using nabiximols (CBD+THC) spray as a medication for pain, four RCT studies concluded a positive effect on their pain while on nabiximols spray compared to placebo [8]. While Rabgay concluded that the evidence is insufficient to determine CBD’s efficacy in pain, Boyaji found it challenging to recommend CBD’s use in chronic pain. Access to pure CBD alone is the main reason for these conclusions.

Some studies showed promising evidence to support the safety of CBD. A review of 16 RCTs conducted by Mücke et al. in 1750 adult participants with neuropathic pain showed that cannabis-based medicine might help achieve >50% pain relief (primary outcome) compared with placebo [16]. It also increases nervous system adverse reactions, including psychiatric disorders, in 17% of participants [16]. In addition, Fisher et al., in their review, made a recommendation to delay the use of cannabis until adolescence, avoid highly potent and widespread use, and prevent smoking cannabis from reducing its adverse effects like cardiovascular, physical, neurocognitive, psychosis, and mental problems [17]. In comparison, it can be deduced that proper dosage and route of administration are essential to gain the maximum effect from CBD use. CBD for pain relief still has a long way to be fully established, but the majority of studies possess promising outcomes. Therefore, formulation of the safety standard used for CBD could be a possibility soon if the growing evidence from more studies points to the efficiency and safety of CBD. Weighing the benefit versus the risk, backed by evidence, is a crucial step. The outcome of each study mentioned above can set a new playing field for pharmaceutical companies for drug development to explore and investigate using clinical trials in a large sample population.

Chronic pain is persistent pain for more than or equal to three months in duration. It has been a complex issue, especially with its variable causes, the complexity of the associated symptoms, and opioid dependence [26]. Scientists and researchers are looking for alternative means to address chronic pain using more substantial evidence from clinical trials and observational studies. In an RCT done by Lichtman et al., nabiximols (THC+CBD) oromucosal spray was used as an adjunct treatment in 291 patients with advanced cancer and chronic pain on opioids [22]. The primary endpoint is the improvement of the average pain Numerical Rating Score (NRS) from baseline. NRS is calculated as the median difference between groups, which showed a positive value of 3.41% (95% CI: 0.00%-8.16%; p=0.0854) in favor of the nabiximols group. No statistical significance was noted in the primary outcome [22]. However, there is improvement in other aspects such as Subject Global Impression of Change (SGIC), Physician Global Impression of Change (PGIC), and Patient Satisfactory Questionnaire (PSQ) from nabiximols compared to the placebo group [22]. Clinical improvement was noticed in the nabiximols group, though not statistically significant.

On the other hand, Capano et al. did a prospective cohort study (with 97 participants) about the effect of CBD hemp extract on patients with chronic pain taking opioid medication [7]. The primary outcome showed that at week 8, 50 out of 94 (53.2%) had decreased their opioid medications [7]. The secondary outcome reported that 89 (94%) improved quality of life as measured by pain and sleep-related open-ended questions. In a similar cross-sectional survey with 1087 patients, Boehnke et al. determined the relationship between the route of administration, CBD content, and timing of use in managing chronic pain [6]. It was noted that the younger population uses inhalation while older people prefer the non-inhalational route. The mixed (inhalation + non-inhalation) route is preferred (45% of respondents), and this is attributed to the tailored pain relief experienced [6]. The content of CBD and timing of use showed that CBD with sedation effects (Indicas) is usually taken at night. Boehnke et al. reiterated in this study that subgroups in the sample population are essential in analyzing the results of CBD use [6]. These two observational studies mentioned above hold decent evidence of the positive effect of CBD on chronic pain, like reduced opioid intake and improved sleep. However, there is a challenge for patients to report the actual outcome observed because health insurance covers opioid medication but not CBD. Therefore, there is fear on the patients’ part about CBD’s availability after research and the financial cost they would have.

Management of chronic pain poses many challenges. With the crisis of opioid use and dependence, medical providers and the government need to work hand in hand to urgently find alternatives to the treatment of chronic pain, whatever the reason may be [27]. More studies and research are rolling in to provide evidence-based solutions to the current crisis. However, more minor studies are focused on using pure CBD products, which are nonintoxicating. As this systematic review proceeded, challenges and questions about CBD use in chronic pain were revealed. More published reviews and studies show promising results for the effect of CBD on pain relief, yet there is difficulty in making any recommendations. Regulations and categories of CBD need to be updated to make clinical trials easier. When evidence of CBD’s pain relief is fully recognized, guidelines need to be applied to the health insurance business to lessen its financial burden on the patient. An opioid is covered by most insurance, while CBD is not. In addition, good-quality and affordable CBD products should be available once everything is in place.

Limitations

A systematic review of the efficacy and safety of pure CBD products was initially planned, but there are limited studies and articles available. Clinical trials on CBD are also scarce because it is relatively new and obtaining a good quality product is still a problem. In addition, it was difficult to find studies that focused on CBD alone since THC is often mixed with it. Access to free full-text papers is constrained as some good-titled articles need payment to gain access. The language of the publication is also limited to English. Although the business of medical marijuana and CBD dispensaries is old, most countries worldwide are still regulating it to make it legal. Hence, there is a limitation in conducting studies on CBD products.

## Conclusions

This systematic review aims to determine the status of the efficacy and safety of CBD on chronic pain. Although CBD and THC come from the same cannabis plant and have almost identical chemical structures, they attach differently to a CB1 receptor, eliciting different effects. One is psychoactivity, prominent in THC but not in CBD. Regulations of CBD worldwide are still a highly debatable issue. Even states and territories of the U.S. differ on cannabis regulations, mainly because of the possible risks outweighing the benefits and the availability of pure CBD products. CBD for chronic pain as an adjunct medication has gained popularity since it is easier to access and has less physician guidance. Some observational and clinical studies lead to CBD’s effectiveness and safety in chronic pain; however, the evidence is not strong enough to obtain a proper recommendation. It is essential to know that pure CBD extract is a strong candidate as an alternative to opioid medication since it is nonintoxicating and dependence is less. This systematic review can benefit other researchers and even ordinary people eager to know the latest updates on CBD research on chronic pain. In the future, clinical trials should focus more on using pure CBD extract to treat chronic pain to attain evidence to properly recommend CBD in the health insurance sector so that patients may benefit to the full extent.
